# Vegetable Oils as Alternative Solvents for Green Oleo-Extraction, Purification and Formulation of Food and Natural Products

**DOI:** 10.3390/molecules22091474

**Published:** 2017-09-05

**Authors:** Edinson Yara-Varón, Ying Li, Mercè Balcells, Ramon Canela-Garayoa, Anne-Sylvie Fabiano-Tixier, Farid Chemat

**Affiliations:** 1 Laboratoire GREEN, Université d’Avignon et des Pays de Vaucluse, INRA, UMR408, GREEN Extraction Team, F-84000 Avignon, France; anne-sylvie.fabiano@univ-avignon.fr (A.-S.F.-T.); farid.chemat@univ-avignon.fr (F.C.); 2 Department of Chemistry, University of Lleida, Av. Alcalde Rovira Roure 191, 25198 Lleida, Spain; balcells@quimica.udl.cat (M.B.); canela@quimica.udl.cat (R.C.-G.); 3 Department of Food Science and Engineering, College of Science and Engineering, Jinan University, Guangzhou 510632, China

**Keywords:** vegetable oils, alternative bio-based solvents, green oleo-extraction, natural products, polar paradox, solvent-solute simulation

## Abstract

Since solvents of petroleum origin are now strictly regulated worldwide, there is a growing demand for using greener, bio-based and renewable solvents for extraction, purification and formulation of natural and food products. The ideal alternative solvents are non-volatile organic compounds (VOCs) that have high dissolving power and flash point, together with low toxicity and less environmental impact. They should be obtained from renewable resources at a reasonable price and be easy to recycle. Based on the principles of Green Chemistry and Green Engineering, vegetable oils could become an ideal alternative solvent to extract compounds for purification, enrichment, or even pollution remediation. This review presents an overview of vegetable oils as solvents enriched with various bioactive compounds from natural resources, as well as the relationship between dissolving power of non-polar and polar bioactive components with the function of fatty acids and/or lipid classes in vegetable oils, and other minor components. A focus on simulation of solvent-solute interactions and a discussion of polar paradox theory propose a mechanism explaining the phenomena of dissolving polar and non-polar bioactive components in vegetable oils as green solvents with variable polarity.

## 1. Historical Evolution of Vegetable Oils Applications

At the beginning of human civilization, animal fats like butter produced from milk of horses, goats, sheep and cattle were probably used instead of vegetable oils before the discovery of oil pressing and extraction from olives or seeds afterwards [1]. In Egypt, Mesopotamia, Greece and Rome, olive oil became the primary source of oil for cooking or use as a condiment. Nevertheless, prior to their use of olive oils, the Egyptians extracted oil largely from radishes or flax seeds, and the Mesopotamians extracted it primarily from sesame seeds [1,2]. Likewise, archaeological and documentary records evidenced that sesame oil competed with the olive oil in the Mediterranean basin and India from at least 1137 BC [3]. Ancient Egyptians firstly produced infusions of medicinal or aromatic plants in vegetable oils as solvents for therapeutic, nutritional, aesthetic and spiritual purposes. They used vegetable oils in the formulation of cosmetics providing emollience, moisturizers and grooming, or acting as solvents and vehicles to carry other agents [4]. They also invented frying in oils or fats for the preservation of meat and vegetables around 2500 BC [5]. In addition, recorded pictorial documents have pointed that edible vegetable oils were used as lubricating liquids as well by Egyptian pyramid builders when rolling large pieces of rocks on wooden rollers [6].

Subsequently, other ancestors such as those from ancient Greece, where wild olives were probably native, had mastered infusion of olive oils with flowers or herbs for beauty and medical treatments. The ancient Romans spread the application of such oils into ordinary lives (e.g., wall coverings, personal care, etc.) after they took over control from the Greeks. European civilizations also used oils or fats infused with aromatic plants such as cinnamon and clove to prevent plagues in the medieval period. In relation to the ancient American civilizations, oils pressed from chia and amaranth seeds were used as body emollients rather than frying. The Aztecs produced groundnut oil in South America long before the arrival of European settlers in the 15th century [3]. Other edible oils like palm oil, originated in West Africa, were spread to the Americas and Indonesia in the 16th century, but the biggest change occurred in the global lipid consumption over the last centuries. In the 18th century, the enfleurage developed in Grasse (France) used odourless animal fats to capture fragrant substances exuded by plants, which led to the production of high-grade concentrates for delicate floral botanicals such as jasmine and tuberose. However, the fats used in this out-dated method were superseded extensively by petroleum-based solvents (e.g., hexane, benzene, etc.) in the 19th century.

In addition to those mentioned above, Asians, especially the Chinese, have also made remarkable contributions to the use of animal fats and vegetable oils. According to historical records, the use of animal fats mostly for cuisine and sacrificial ceremonies remounts to the Xia-Shang-Zhou Dynasties (2070–1600 BC), and lasted till the first appearance of vegetable oil from primitive pressing or extraction in the Western Zhou Dynasty (1046–771 BC). However, the variety of vegetable oils was very limited, i.e., most were sesame oils at that time. This situation got better in the Song (960–1279 AD), Ming (1368–1644 AD) and Qing (1612–1912 AD) dynasties, during which the development (e.g., oil-bearing plant cultivation, extraction and application) of vegetable oils flourished as never before. Indeed, during the Song period the gradual encouragement of Buddhism and vegetarianism initiated by temporal scholar-bureaucrats pushed these uses. Besides, the legendary Chinese namely Shennong experienced oils or fats infused with more than hundreds of herbal medicines as long ago as 2800 BC. His oral traditions written between about 300 BC and 200 AD had been collected, sorted out and edited in “Divine Farmer’s Materia Medica” [7]. From this codex to the following classic “Compendium of Materia Medica” written by Li Shizhen (1518–1593) [8], the application of infused oils had gone through a thousand years’ development and evolvement. Until now, the acupuncture and moxibustion assisted by oils infused with medicinal plants have still shown their miraculous effectiveness in traditional Chinese medicine.

Nowadays, vegetable oils play an important role in our ordinary diet, consumed directly in their refined or virgin forms, or via many food industrial products. Such oily products could also be used as ingredients or components in many other fields such as cosmetics, nutraceuticals, paints, lubricants and biodiesel. Therefore, the restudy of vegetable oils as alternative solvents in the 21st century will prompt the development of efficient strategies for their promising future applications such as extraction, purification and formulation.

## 2. Vegetable Oils Are Not Only Triglycerides

### 2.1. Major Components in Vegetable Oils

Vegetable oils are usually produced from plant seeds or fruits (e.g., rapeseed, sunflower, olive, etc.), simply by pressing and/or solvent extraction. They are considered nonpolar and lipophilic systems whose composition is highly variable and complex, depending on their origin, quality and producing methods. Vegetable oils have a relatively high flash point, a selective dissolving power and an attractive price (≈0.5–5 €/kg) [9].

**Figure 1:**
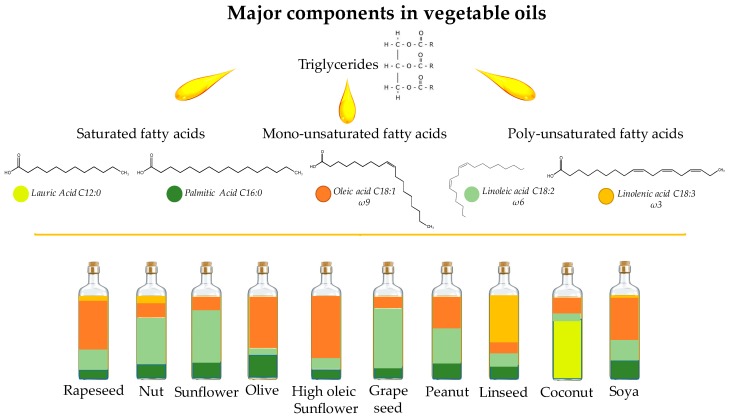
Major components in different vegetable oils.

Triglycerides (Figure 1), which are composed of three fatty acid molecules esterified to one glycerol molecule, are the main building blocks of vegetable oils (95–98%). The characteristics of triglycerides are determined by the types, proportions and positions of fatty acids on the glycerol backbone. The fatty acid composition of triglycerides in vegetable oils varies depending on varieties, cultivations, agronomic, and climatic conditions. Typically, even-numbered carbon atoms dominate the fatty acid chains’ length in vegetable oils due to their bio-syntheses pathways. According to the saturation degree of fatty acids, they can be generally classified into saturated, mono- and poly-unsaturated fatty acids, which may have different configurations, resulting in different physical and chemical properties.

### 2.2. Minor Components in Vegetable Oils

**Figure 2:**
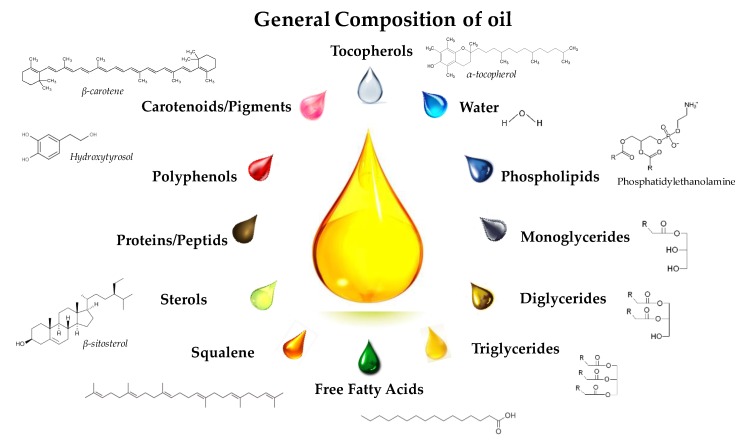
Minor components in vegetable oils.

In addition to triglycerides, the presence of minor components (less than 5%) in vegetable oils (Figure 2) cannot be ignored due to their interesting biological properties and nutritional values for pharmaceutical and nutraceutical industries, particularly in virgin oils [10,11]. They can be divided into two types: glycerolipids such as mono- and diglycerides, phospholipids, and non-glycerolipids including sterols, tocopherols/tocotrienols, free fatty acids, vitamins, pigments, proteins, phenolic compounds, water, etc. [12,13]. Although some undesirable minor components must be removed by most consumers and food manufacturers during the refining processes, there is no completely efficient and selective process, which will give rise to consequent colloidal structures formed by residual minor components in oils [14].

#### 2.2.1. Mono- and Di-Glycerides

Partial glycerides, i.e., mono- and diglycerides, are mono- or diesters of glycerol with fatty acids (Figure 2), which are well known for their emulsifying properties. They are authorized food additives (E471), which can be obtained by partial hydrolysis of triglycerides during the extraction of vegetable oils. They are naturally present in virgin oils due to the action of endogenous lipases in fruits or oilseeds. The enzymatic hydrolysis leads to the formation of both partial glycerides and free fatty acids, in which partial glycerides account for 1–6 wt % depending on oil types, for instance, the concentration of di glycerides ranges from 1 to 2.8% in the virgin olive oil and monoglycerides present at much lower levels (less than 0.25%) [15]. The content of diglycerides in olive and palm oils is higher than that in virgin oils. Moreover, the neutralization and washing stages in the oil refining processes cannot eliminate partial glycerides in their refined forms. Mono- and diglycerides are amphiphilic molecules due to the presence of free hydroxyls, which give them a certain hydrophilicity with a hydrophilic-lipophilic balance (HLB) of about 3.4–3.8 and 2–5, respectively [16,17].

#### 2.2.2. Phospholipids

Phospholipids are considered polar lipids present in plant cell membranes, which co-extract with neutral triglycerides during squeezing or solvent extractions of both seeds and waste virgin oils using hexane. The main plant sources are soybean, rapeseed and sunflower. Most phospholipids are removed in the degumming step of the oil refining process in order to avoid foaming or browning during technological processing operations for edible oil uses. Phospholipids consist of a glycerol backbone, two fatty acids and one polar phosphate group associated itself with an amino alcohol (choline, ethanolamine, serine) or a sugar (inositol) as shown in Figure 2. Lecithins (phosphatidyl choline) are authorized food additives (E322) with wide applications, specifically in baking industry. This feature is because of their significant surfactant effect (HLB ≈ 2–8) [16,18]. Moreover, lamellar structures can be formed in the presence of phospholipids and other surface active compounds such as sterols, which are hard to characterize.

#### 2.2.3. Free Fatty Acids

Free fatty acids are generated from the hydrolysis of triglycerides or phospholipids. They are usually eliminated during deodorization and physical refining processes due to their impacts on foaming and the smoke point of oils. Normally, virgin and refined vegetable oils or fats have free fatty acid contents of less than 5% and 0.1%, respectively. However, their contents are higher when oils have high mono- and diglyceride percentages. Free fatty acids with a low HLB value of approximately 1.0 can assist the formation of reverse micelles so as to stabilize water-in-oil emulsions. Most fatty acids of natural origin have an alkyl chain comprising between 4 (C4) and 22 (C22) carbon atoms. The most common unsaturated fatty acids are C16 and C18. Short or medium chain fatty acids are normally recognized below these lengths whereas fatty acid chains with more than 18 carbons are often deemed to be long chain fatty acids [19].

#### 2.2.4. Sterols

Apart from the very low content of cholesterol in vegetable oils, plant sterols, namely phytosterols, are not only some of the important minor compounds with considerable biological activities [20,21], but also the predominant compounds in the unsaponifiable fraction of vegetable oils. In general, β-sitosterol is the most abundant phytosterol in vegetable oils, along with variable amounts of other sterols (e.g., stigmasterol, campesterol, ∆^5^-avenasterol, brassicasterol, etc.) depending on the oil type [22]. Phytosterols are affected by several oil refining processes, in which deodorization can significantly reduce the total sterols due to distillations and esterifications of free sterols [11]. Since phytosterols are rather hydrophobic and poorly soluble in fats, they are usually esterified to make them liposoluble for easily processing handling in the food industries [23].

#### 2.2.5. Tocopherols and Tocotrienols

Tocopherols and tocotrienols are well-known vitamin E compounds, which are vital for enhancing the oxidative stability of vegetable oils because of their strong inhibitory effects against lipid oxidation [24,25]. Naturally, tocopherols are presented as free alcohols while tocotrienols are in esterified forms [26]. There are four common types of tocopherols and tocotrienols (α, β, γ and δ), in which the number and the position of methyl groups on chromanol skeletons are different. The content of tocopherols and tocotrienols in different types is dependent on the oil type, which may also be influenced by their unintentional removal during the oil refining, especially in the deodorization [27,28].

#### 2.2.6. Water

A water content of less than 0.05% is generally controlled in commercial vegetable oils, which may change during preservation periods after opening as moisture absorption or loss happen according to the storage environment [29]. Since water is known for its immiscibility with oils, it can probably provide oil-water interfaces in vegetable oils where endogenous or exogenous surface active compounds with low HLB values migrate, preferably concentrate to form and to stabilize association colloids such as reverse micelles and lamellar structures [30]. These energetically preferential structures enable the coexistence of both hydrophilic and lipophilic compounds, which may inspire a novel solution for the extraction of water-soluble compounds in vegetable oils.

#### 2.2.7. Other Minor Compounds

A wide range of phenolic compounds were reported at varying levels in vegetable oils (particularly in olive oils) depending on the oil type, ripeness, storage and processing conditions [31]. These polar compounds possessing free radical scavenging activity are important for the oxidative stability of the polyunsaturated fatty acids in vegetable oils. They tend to stay in the polar region of association colloids. Squalene is a triterpenoid hydrocarbon of thirty carbons, which presents in vegetable oils at a very low level [32]. It can be used in healthy foods and its hydrogenated form can be applied as a moisturizing agent in cosmetics. Pigments such as carotenoids in most virgin oils appear as a conjugated system with alternated double bonds in a long chain of forty carbons. They can be classified in nonpolar (e.g., α- or β-carotene and lycopene) and more polar carotenoids with oxygen at one or both end groups. Chlorophylls usually mask the colour of carotenoids in freshly pressed virgin oils, whose variable contents are determined by the oilseed maturity and the oil extraction method. Furthermore, the presence of proteins and peptides has also been investigated as minor components in virgin oils, which has attracted increasing concerns on the stability and the allergenicity of oils [33,34]. Besides, trace quantities of other components such as metals, hydrocarbons and amphiphilic lipid oxidation products are deleterious to oil quality so that they should be reduced to the minimum [22].

## 3. Why Choose Vegetable Oils as Alternative Solvents?

Although no official standards exist so far for the alternatives to petroleum-based solvents, the ideal properties of a green solvent to minimize environmental impacts during its complete life cycle have been summarized [35]. These bio-sourced or agro-based solvents should be non-volatile, safe, easy to regenerate and economically viable. Most importantly, they must have considerable dissolving power and selectivity. Hence, vegetable oils may have the potential of being a green alternative solvent due to their good consistency with ideal solvent properties.

Empirical infusion or maceration of medicinal and aromatic plants in vegetable oils as solvents dates back to ancient civilizations as already mentioned. In recent years, edible commercial vegetable oils have been successfully enriched or aromatized with bioactive compounds from herbs, spices or other plant materials in order to improve their nutritional values and organoleptic qualities, and to prolong shelf-life as well. The resulting oils can be defined as “flavoured oil” or “gourmet oil” named as the plant extracted. As the consumption of these products has drawn more interest due to their particular capacities for the prevention of diseases through a healthy diet, their versatility, convenience, and wide range of tastes have made them diffuse rapidly among traditional and non-traditional consumers across many countries in the world. Therefore, it is meaningful to explore more on their characteristics and applications as they are gaining an increasing popularity in diets, which can help to establish a sound labelling standard for strict regulations [36]. Nowadays, various enriched or aromatized oils are available in the market as seasonings. Moreover, considering that the solvent is often the major ingredient of a formulation, a reaction, or an extraction [37], vegetable oils are excellent alternatives for the substitution of petroleum solvents, such as hexane, in the extraction of bioactive compounds, and finally, they allow a better integration in the formulation of products. Table 1 shows different applications of various vegetable oils as solvents in natural product extractions, enriched oil preparations, and the formulation of products with applications in food and cosmetic industries.

As summarized above, most laboratory studies have proved the good dissolving power of vegetable oils, as well as better organoleptic quality and oxidative stability of their enriched forms. Since using vegetable oils as solvents in the direct extraction of carotenoids from shrimp by-products and microalgae has been successfully applied in several studies [38,45,48,49,50,53], vegetable oils have proved their effectiveness in retaining high concentration of carotenoids under optimal conditions without any loss or degradation of carotenoids, or changes in the fatty acid profiles of the oil. Moreover, vegetable oils are usually enriched or flavoured with antioxidants or aromatic extracts from plants or by-products, which are obtained by means of traditional (infusion, one-step solvent extraction, etc.) or innovative methods such as ultrasound and supercritical CO_2_ using petroleum-derived organic solvents. This is consistent with a previously reported finding indicating that vegetable oil was a remarkably good solvent for a wide variety of food aroma compounds [67,68]. Although these organic solvents have been evaporated afterwards in enriched oils, it has also drawn an increasing concern on their safety in food-related applications and environment. In addition, the selectivity of vegetable oils is dependent on their types and components inside, resulting in variable extraction efficiency and enrichment factors. For vegetable oil solvents targeting to six major aroma extracts (i.e., linalool, estragole, eucalyptol, trans-anethole and limonene) from basil, refined sunflower oils was theoretically and experimentally proven as the most suitable solvent among ten vegetable oils [68]. Notwithstanding, such sunflower oil was not the optimal in the extraction of phenolic compounds from olive leaves as compared to its virgin form and several other vegetable oils like avocado, flaxseed and castor oils [69]. The extraction yield of total phenolic compounds was found to be more related to the type of vegetable oils and endogenous amphiphilic minor compounds rather than the polyunsaturated degree of vegetable oil triglycerides.

### 3.1. How to Select a Good Solvent?

A solvent is defined as “a liquid that has the property to dissolve, dilute or extract other materials without causing chemical modification of these substances or itself. Solvents are able to implement, apply, clean or separate products” [70]. These compounds play an important role in great number of unit operations in chemistry and chemical engineering. In fact, nowadays there is no production process in perfume, cosmetic, pharmaceutical, food ingredients, nutraceuticals, biofuel or fine chemicals industries without a solvation step [71]. Solvents can be used as diluents or additives in paints and inks, as cleaning agents or solvents for syntheses and extractions. From a macroscopic point of view, a solvent is a continuum characterized by physical constants (e.g., boiling point, melting point, vapour pressure, relative permittivity, thermal conductivity, surface tension, density, viscosity, refractive index, etc.) [72], whereas from a microscopic point of view, it is a discontinuum that consists of individual solvent interacting molecules characterized by molecular properties (e.g., dipole moment, electronic polarizability, hydrogen bond donor or acceptor character, electron donor or acceptor character, etc.). These different properties are at the origin of solute/solvent interactions during the solvation process.

The problem with most commonly used solvents is their negative impacts on health, safety and environment (HSE) as most solvents currently available in the world market come from the petrochemical industry and are volatile organic compounds (VOCs), such as the example of hexane used for oil extraction. In 2009, the global market of chemical compounds represented around 100 billion dollars, in which only 3% of these chemicals were obtained from renewable resources, after chemical processing, fermentation or enzymatic conversion. This share is predicted to attain around 15% by 2025, when the global market is estimated to reach 3000 billion dollars [73]. With the geopolitical environment (increased oil prices and decrease of reserves), societal demand for more sustainable products and the arrival of the new regulations and guidelines (e.g., REACH, etc.), much interest has been generated to the development of new eco-friendly ways of replacing conventional solvents. In other words, alternatives to petrochemical solvents have to fulfill the principles of Green Chemistry.

**Figure 3:**
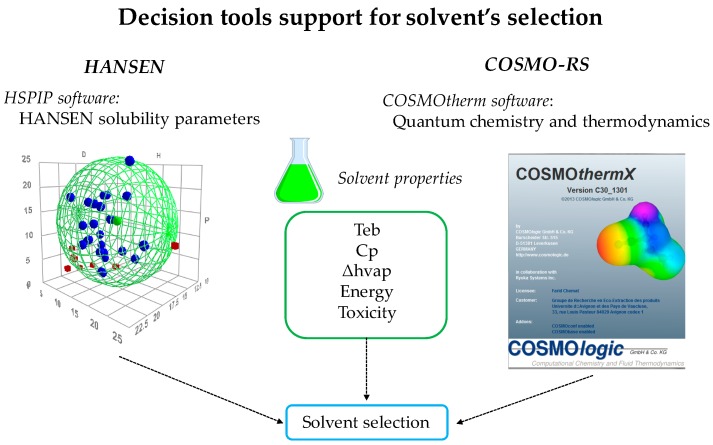
How to select a good and green solvent?

Replacing one solvent by another does not necessarily mean eliminating all the hazards and issues related to the implementation of a process. Indeed, the modification of a process is generally associated with new risks. Precautions should therefore be taken into account in the selection of an alternative solvent as for physicochemical, environmental or sanitary criteria, the eco-compatibility of the process and the price of the solvent, but also techno-economic criteria related to the properties of the solvent like dissolving power and energy consumption [74]. The dissolving power is a key criterion that can be evaluated using various methods such as the Kauri-butanol index, Kamlet-Taft scale, or Hildebrand and Hansen solubility parameters (HSP), but also thanks to a much more powerful tool, COnductor like Screening MOdel for Real Solvents (COSMO-RS), that can be used as a real decision tool for the choice of alternative solvents (Figure 3). On the other hand, pharmaceutical companies such as GSK [75], Sanofi [76], AstraZeneca [77,78] and Pfizer [79] developed their own solvent selection guides that provide technical data and clear instructions for the development of more sustainable processes. This allows the involvement and commitment of industries in the investigation on greener alternative solvents and proves their concern for the integration of sustainable development approaches.

To sum up, an ideal alternative solvent must fulfill the following requirements: (a) does not emit VOC; (b) be of low toxicity for humans; (c) have a limited impact on environment (be eco-friendly); (d) be obtained from renewable resources; (e) have a high dissolving power; (f) be easy to recover; and (g) does not change the process set-up significantly. To this end, new technologies such as solvent-free methods, aqueous formulations or alternative solvents, appear to be good candidates. Among these solutions the use of greener solvents, such as bio-based solvents, constitutes one of the most important alternative routes for the substitution of petrochemical solvents. The term “sustainable” or “green” is used to describe different types of solvents including the ones that are produced from biomass feedstock and eco-friendly petrochemical-based solvents that are non-toxic and/or biodegradable [80].

There are seven classes of solvents generally claimed as “green” solvents [81], including (1) bio-based solvents (from renewable resources), (2) eco-friendly (good HSE profile), (3) water (renewable and non-toxic), (4) liquid polymers (non-volatile, biocompatible, non-toxic), (5) fluorinated solvents (non-flammable and non-toxic), (6) ionic liquids/eutectic mixtures (non-volatile, thermally stable. e.g., imidazolium salts, choline acetate) and (7) supercritical fluids (CO_2_, inert, recyclable, non-flammable, non-toxic). It is worth noticing that the greenness of some solvents is questionable with regard to toxicity (e.g., ionic liquids) or biodegradability (fluorinated solvents and silicones). 

In this context, vegetable oils used as solvents belong to the bio-based solvent class, which have the advantage of offering a positive impact on the environment and health (no emission of volatile organic compounds, biodegradable and non-toxic). Vegetable oils are non-polar lipophilic systems whose composition varies considerably according to their origins, quality and methods from which they were obtained. Commonly used in cosmetics or in food industry, they can also be applied to extraction field as for example to achieve bioactive phytochemicals from natural resources. Vegetable oils have successfully been used as solvents for the extraction of carotenoids from by-products of crustaceans [38,49,53] or from fresh carrots [82], and also for the extraction of aromas from basil [68]. Likewise, vegetable oils have been enriched with diverse products such as aromas, polyphenols, antioxidants and pigments, which confer to the oils better oxidative stability and notable organoleptic qualities. Recently, extractions with vegetable oil solvents have been associated with various innovative technologies: ultrasound, microwave and supercritical CO_2_. The extraction of carotenoids from fresh carrots assisted by ultrasound showed similar yields to that obtained with hexane, under optimized conditions [82]. Sunflower oil as the solvent allowed the simplification of the extraction process, by the absence of the evaporation step or solvent separation. This greener technique also offered significant advantages in terms of cost, time and energy. Otherwise, supercritical carbon dioxide extractions of carotenoids using various vegetable oils as co-solvents have also been widely studied. The beneficial role of the addition of vegetable oil co-solvents was demonstrated, as it improved the solvation performance and prevented the degradation of target carotenoids during the extraction. In addition, a work performed by Chevereau [83] determined that using vegetable oils as solvents and microwave as the extraction method provided an improvement in the extraction efficiency of bioactive extracts without degradations or contaminations of the oil. The improved dissolving power (high yields, optimized organoleptic properties) of vegetable oils by association with ultrasound and microwave was also presented in the work of Rossignol-Castera [84]. As the application of vegetable oils as alternative solvents has emerged in many areas, a good investigation of various components in vegetable oils is necessary for better understanding their underlying dissolving mechanism, which could lead to a green strategy for extraction techniques.

### 3.2. Can Nonpolar Vegetable Oils Be Good Solvents for Polar Antioxidants?

**Figure 4:**
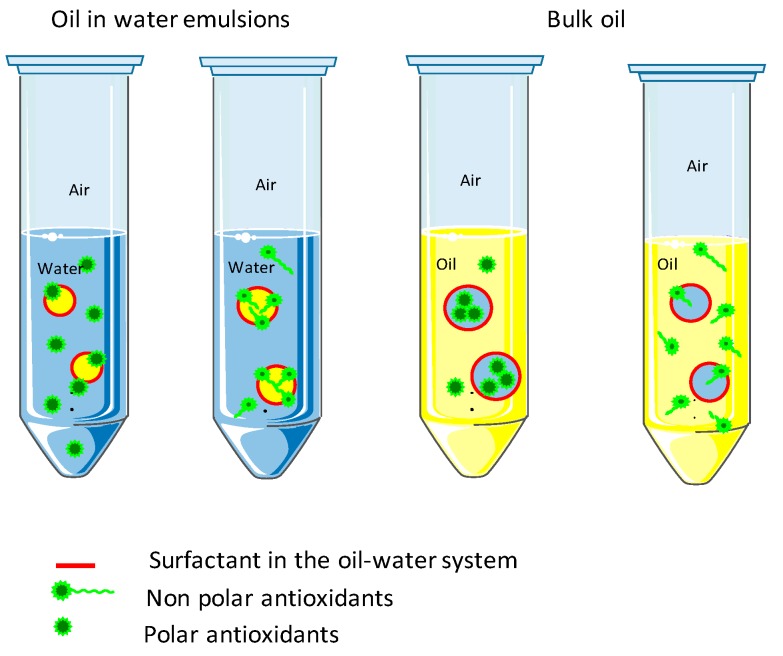
Polar paradox theory.

Knowledge developed during the last two decades helped us to gradually find out that the effectiveness of antioxidants in the lipid oxidative inhibition is depending on both chemical reactions and physical molecular orientations, self-assembled microenvironments in particular. According to the “polar paradox” breakthrough [85,86], polar or hydrophilic antioxidants with high HLB values are more effective in non-polar media such as vegetable oils with a low surface/volume (LSV) ratio whereas nonpolar or lipophilic antioxidants with low HLB values tend to be more effective in more polar media such as oil-water emulsions with a high surface/volume (HSV) ratio. This hypothesis was turned from an empirical observation into a putative theory thanks to the explanation by the interfacial phenomenon [87,88]. The previously believed assumption on oxidation in vegetable oils as homogeneous media was questioned because oil is more polar than air. Since polar antioxidants, amphiphilic molecules and trace amounts of water coexist in whatever vegetable oils, they will aggregate to form different types of colloidal associations (e.g., reverse micelle, lamellar structure, etc.), which are considered oxidation sites [22]. The polar antioxidants distributing at the oil-air interface are indeed preferentially located at the oil-water interface of colloids, hence perform more effective in oxidative inhibitions than nonpolar ones that are dissolved in the lipid phase (Figure 4). The polar paradox was found not applicable for the overall concentration range of antioxidants as more evidences have indicated a non-linear cut-off effect for nonpolar antioxidants that are more effective below a critical concentration than polar antioxidants in vegetable oils because the solubility effect outweighs the interfacial phenomena. The reversed trend occurred after this critical concentration is the polar paradox case, which depends more on the micellar effect [89].

Paradoxes and inconsistencies are constantly emerging. Supramolecular chemistry as a new paradigm may shed light on lipid oxidation and antioxidant effectiveness now by reconsidering molecular shape, supramolecular interactions and the distribution of polar and nonpolar regions [90]. In vegetable oils, water inside or from atmosphere and other polar compounds are located in the core of reverse micelles. A depletion layer almost without surfactants exists next to the core and the outermost monolayer was comprised of surfactants, which solubility could be increased with the help of co-surfactants (e.g., short or medium chain alcohols, glycerol, sorbitol, etc.). These self-assembled micelles are considered as thermodynamically stable nano-reactors, which have different sizes and allow the dissolution of a desirable amount of polar and non-polar compounds [91]. Hydrophilic lipophilic balance (HLB) is empirically used to describe the antioxidant solubility in lipid systems. Endogenous or exogenous surfactants with low or intermediate HLB values, for instance, phospholipids, free fatty acids, mono- and diacylglycerides, are more favoured to form and to stabilize reversed micelles [22]. Traces of these minor compounds in virgin or refined vegetable oils play a vital role in micellization and further lipid preservation. Nonetheless, there are still other influencing factors (e.g., pH, log P, double bond position in unsaturated fatty acids, acyl chain length, ionicity and packing parameter of surfactants, etc.) for antioxidant effects and micelle stabilization in lipid systems, among which water activity is of paramount importance.

Inspired by the paradoxical behaviour of polar antioxidants in bulk lipids, vegetable oils can not only be the solvents for the lipophilic antioxidants in light of the “like dissolve like” principle. The self-assembled micellization in vegetable oils may help to develop an in-site direct extraction of hydrophilic antioxidants [69], where extraction efficiency, selectivity and enrichment factors are variable depending on the oil type and composition, as well as surfactant type and dosage [92]. The spherical reverse micelles could be formed by a simply intentional addition of food-grade surfactants and proved by small angle X-ray scattering (SAXS). However, the appropriate dosage of surfactants should be further evaluated to make sure their critical micellization concentrations and interactions in a supramolecular microenvironment. Likewise, this novel extraction may provide a more efficient strategy for the lipid oxidative prevention in further formulations. 

### 3.3. Which Kind of Molecules Can Be Solubilized in Vegetable Oils?

Determining the different molecules that can be solubilized into vegetable oils seems relatively easy if we know the polarity of the target molecules. However, there are some considerations in the prediction of the dissolving power of vegetable oils such as their supramolecular complexity, various extraction methods, target solute, acylglycerol and fatty acid composition, etc. Today, one way to predict the solubilization of one molecule into different solvents including vegetable oils, avoiding the waste of time in resorting to trial and error experiments, is the use of efficient predictive tools, among which the most powerful one is the COSMO-RS that we have currently paid particular attention on.

#### COSMO-RS Approach

**Figure 5:**
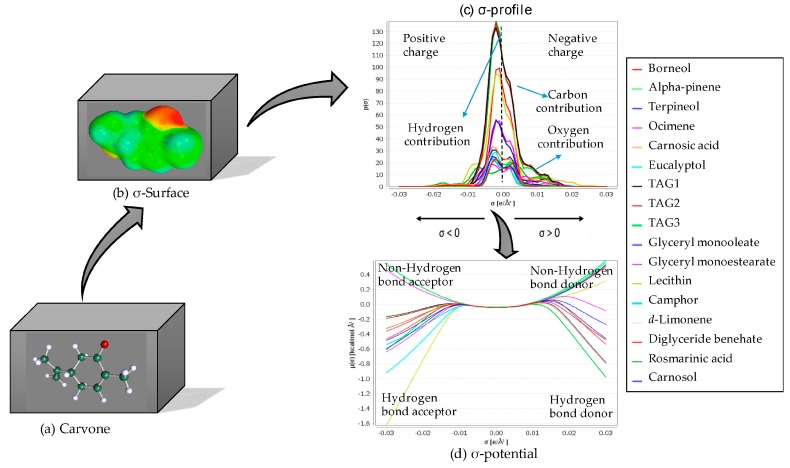
COSMO-RS to select ideal solvent for dissolving a suitable compound. (**a**) Carvone (solute molecule); (**b**) σ-surface; (**c**) energies of local surface interactions between σ-profiles of carvone and solvents; (**d**) σ-potentials of carvone and solvents.

The significant improvement in computational power and sophistication of recent algorithms led to the possibility to extensively use quantum descriptors of the solvent effect. Cartier et al. showed that quantum chemistry provides a more accurate and more detailed description of electronic effects than empirical methods [93]. Thanks to a combination of a dielectric continuum solvation model and a thermodynamic treatment of the molecular interactions, Klamt developed a general approach in which a solvent can be treated in the liquid state [94]. In the first step of the COnductor-like Screening MOdel, usually denoted as COSMO, the solute molecule is considered to be embedded in a cavity that is surrounded by a virtual conductor. In this environment the molecule induced a polarization charge density on its surface depicted on the σ-surface. During the quantum calculation, the solute molecule is converged to its energetically optimal state in the conductor with respect to its electron density and geometry. In the second step, with the extension RS for “Real Solvent”, a combination of an electrostatic theory, COSMO-RS, with the statistical thermodynamics treatment of interacting surfaces is used. The spatial distribution of the polarization charge σ of the molecule is then converted into a surface composition function (σ-profile). This σ-profile provides information about the molecular polarity distribution (Figure 5). 

The thermodynamics of molecular interactions is used to calculate the chemical potential of the surface segment (σ-potential) using COSMOthermX program (version C30 release 14.10). The σ-potential (Figure 5) can be interpreted as the affinity between a solvent and the surface σ via electrostatic interactions and hydrogen bonds. The part of the negative charge of the molecule is located on the right side (acceptor hydrogen bonds) with positive σ values while the part of the positive charges is located on the left side (donor hydrogen bonds) with negative σ-values. Generally, the region σ ± 1 e/nm^2^ is considered to be non-polar or weakly polar. The σ-profile and the σ-potential are used to firstly interpret the affinity of the solvent for surface polarity, and then to understand the interaction between the solute compound and a list of solvents. The thermodynamic properties of the system could finally be estimated. In addition, the software COSMOthermX allows the calculation of the affinity between the solute and the solvent in terms of logarithm of the solubility in mole fractions (log10(x_solub)). The logarithm of the best solubility (i.e., solute and solvent are miscible) is equal to 0 and all other solvents are predicted relative to the best solvent(s).

Despite the fact that vegetable oils have been used as solvents in the extraction of natural compounds, as well as their enrichment with diverse bioactive compounds, predictive methods such as COSMO-RS have not been used to determine the potential of these alternative bio-based solvents. In this context, we have performed the simulation of the solubility of various bioactive products with different polarities in sunflower oils, from lipophilic β-carotene to hydrophilic compounds with higher polarity as antioxidants like hydroxytyrosol. In addition, we have also evaluated the relative solubility of these bioactive compounds in the sunflower oil with surfactants and conventional solvents such as *n*-hexane, ethyl acetate, acetone, ethanol and water. *n*-Hexane, the most commonly employed solvent in the extraction field, was used as the reference. The simulation results showed that the theoretical solubility of the bioactive compounds in general was better in sunflower oil or sunflower oil with 1% *w*/*w* of surfactant than in *n*-hexane. Li et al. performed a direct extraction of phenolic compounds from olive leaves using vegetable oils as solvents and food additives (soy lecithin, diglycerides, unsaturated or saturated mono glycerides) as surfactants [69]. The experimental results showed a significant difference on the extraction yield of phenolic compounds among various refined and unrefined oils, which were mainly dependent on their composition instead of the unsaturation degree of fatty acids. This work concluded that appropriate surfactant additions could significantly improve the extraction yield for refined sunflower oils, where the 1% w/w addition of glyceryl oleate was determined as the optimal. Furthermore, 5% *w*/*w* addition of lecithin performed the best in such enrichments compared with mono- and di-glycerides. The exception in the simulation results was with β-carotene, whose solubility prediction was slightly better in the reference solvent. However, experimental results have shown that vegetable oils are good solvents for the extraction of this sort of natural products. Sun and Temelli [95] carried out a comparison of conventional solvent extraction with supercritical CO_2_ (SC-CO_2_) extraction using canola oil as the co-solvent to extract carotenoids from carrots. It was found that the yield of total carotenoids by SC-CO_2_ extraction with 5% (*w*/*w* of CO_2_) canola oil was higher than that by conventional solvent extraction using hexane or acetone. The triglycerides here can increase β-carotene solubility in supercritical CO_2_, and additionally, vegetable oils help to improve the stability of pigments [96]. Therefore, the use of different vegetable oils as co-solvents in SC-CO_2_ is a new approach for carotenoids recovery with promising results [94,97,98,99,100,101]. Likewise, Li et al. applied sunflower oil as a substitute for organic solvents in carotenoids extraction from fresh carrots using an ultrasound-assisted extraction (UAE) process [82]. This procedure was compared with conventional solvent extraction (CSE) using hexane as the solvent. The results showed that the green UAE process using sunflower oil as the solvent gave the best yield of β-carotene in comparison with CSE.

## 4. Innovative Techniques for Intensified Extraction Using Vegetable Oils as Solvents

Bioactive compounds from plants, algae, yeast and fungi can be extracted by various classical extraction techniques such as maceration, solvent extraction, steam or hydrodistillation, cold pressing, squeezing, and others. Most of these techniques are based on the extracting power of different solvents and the application of heat and/or mixing. Nonetheless, the design of more efficient extraction processes that may address the requirements for process intensification (i.e., faster and more effective energy use, increased mass and heat transfer, reduced equipment size, reduction of processing steps and increase of yields and product quality) has been one of the main research topics in recent years. All safety, sustainability, environmental and economic factors are forcing laboratories and industries to turn to nonconventional technologies and greener protocols. Moreover, due to toxicity and the growing price of fossil resources, the replacement of petroleum-origin solvents is desirable. Consequently, in the last two decades, the use of efficient, innovative and intensified extraction techniques is amenable to automation such as widely used ultrasound, microwave and supercritical fluid assisted extraction. In addition, the combination of ultrasound with conventional Soxhlet extraction [102,103], Clevenger distillation [104] and innovative techniques [105,106,107,108,109,110] has been reported as well [111].

Due to the fact that several special phytochemicals are either destroyed or lost during distillation, or are very difficult to extract resulting in a low yield of essential oils, herbal infused oils are indispensable for obtaining such compounds. Therefore, the solvent property of vegetable oils and their applications with innovative techniques (e.g., ultrasound, microwave, supercritical fluids, etc.) have been explored in bioactive phytochemicals’ extractions from natural bio-resources, which helped to achieve a greener extraction procedure and novel value-added end-products with great potential in food, nutraceutical and cosmetics industries. Table 2 presents some applications of innovative techniques for the extraction of different kind of compounds from various plant materials using vegetable oils as solvents or co-solvents to be enriched with bioactive compounds. In this context, the ultrasound-assisted extraction (UAE) based on Green Extraction and bio-refinery concepts using sunflower oil as the solvent has recently been designed for the extraction of carotenoids from fresh carrots. The extraction yield under optimized UAE conditions was similar to that of conventional extraction using hexane as the solvent [82]. Furthermore, this greener technique also offered significant benefits in terms of cost, time, energy and environment; and showed its potential for an industrial-scale application in relevant fields. In addition, supercritical carbon dioxide extractions of different carotenoids and derivatives using various vegetable oils as co-solvents have also been widely studied. The beneficial role of adding vegetable oils as co-solvent has been demonstrated to enhance the yield, and to avoid the degradation of the target carotenoids during the extraction [95,97,100,101,112]. Apart from this, Chevereau [83] has applied microwaves to improve the extraction efficiency of bioactive extracts in vegetable oils without lipid degradation and product contaminations. Furthermore, Rossignol-Castera [84] proposed a greener extraction procedure intensified by both microwaves and ultrasounds, which could improve the solvent power of oils in order to ensure a good reproducibility of produced extracts with high yields and optimal organoleptic properties. These novel developed techniques have already been put into real production on an industrial scale.

## 5. Conclusions and Future Perspectives

Alternative solvents from renewable resources for extraction, purification and formulation of natural and food products have attracted a lot of attention in recent years. In this sense, this review gives an overview about the use of different vegetable oils as solvents in the extraction of natural products, preparation of enriched oils with various bioactive compounds from natural sources and formulation of products with applications in food and cosmetic industries. Subsequently, this review presents a discussion of polar paradox theory that illustrates the paradoxical behavior of antioxidants in different media and explains the fact that polar or hydrophilic antioxidants with high HLB values are more effective in non-polar media such as vegetable oils whereas nonpolar or lipophilic antioxidants with low HLB values tend to be more effective in more polar media such as oil-water emulsions. Likewise, the use of a powerful and time-saving COSMO-RS simulation tool is presented to combine a dielectric continuum solvation model and a thermodynamic treatment of the molecular interactions, which could help to predict the solubilization of one molecule in different complex solvents like vegetable oils. In addition, the solvent property of vegetable oils and their applications with innovative techniques (e.g., ultrasound, microwave, supercritical fluids, etc.) for the extraction of different compounds from natural bio-resources has also been reviewed. These greener techniques could offer significant benefits in terms of cost, time, energy and environment, and have shown their potential for an industrial-scale application in food, nutraceutical and cosmetics industries. On the other hand, the excellent natural properties of vegetable oils such as global wide availability, biodegradability, low cost and excellent environmental aspects (i.e., low ecotoxicity and low toxicity toward humans), are advantages in the development of value-added products, for instance, the preparation of oils enriched with medium or high polarity bioactive compounds. To achieve this goal, vegetable oils can be modified in order to modulate their polarity and increase their dissolving power in the extraction of polar bioactive compounds with applications in different industrial sectors.

Vegetable oils have been used as solvents for extraction, purification and formulation by ancient civilizations such as Egyptians and Phoenicians, Indians and Chinese, and even Mayas and Aztecs. The challenges launched by the environmental protection and competitiveness of the globalized market strongly require innovations that break away from the past rather than simple continuity. Vegetable oils could be one of the solutions coming from the past and acting as a future of humanity as an ecologic and an economic alternative to petroleum and hazardous solvents, and turning to sustainability in the 21th century. We are totally convinced that this review is only a starting point for future scientific innovations in this new area “vegetable oils as functional ingredients, reagents and solvents” which is already a success story of collaboration between research, industry and education, covering large ecologic and economic applications: perfume, cosmetic, pharmaceutical, food ingredients, nutraceuticals, biofuels, or fine chemicals industries, for processes such as extraction, formulation, purification, pollution remediation, lubrication, and so on.

## Figures and Tables

**Table 1:** Applications of vegetable oils as solvents.

Oil Types	Materials	Extracts	Experimental Remarks	Reference
Soy oil	Crawfish waste	Astaxanthin	Maximal pigment extraction and oil recovery was obtained from a 1:1 ^a^ ratio of oil to crawfish waste.	[38]
Mixture of virgin and refined olive oil	Dry oregano, mature garlic bulbs and rosemary leaves	Essential oils	The flavoured oil with dry oregano and rosemary (2 wt %, dark, nitrogen, 30 min, stirring) are beneficial to organoleptic quality and shelf-life while that with garlic (5:1 ^a^, 24 h, 25 °C, stirring) did not improve the storage stability.	[39]
Sunflower oil	Air-dried, powdered Lamiaceae plants	Antioxidants	Enriched oils contain 0.1~0.5% of organic solvent extracts, in which *Satureja hortensis* L. ethanol extracts performed the best in oil stabilization.	[40]
Olive oil	Rosemary, dry oregano	Phenolic compounds	Total phenol content increased 1.7 and 3.5 times in rosemary and oregano gourmet oils (5 wt %, 24, 48, 72 h agitation, dark), which have superior oxidative stability and consumer acceptability to the origin oil.	[41]
Vegetable oils	Olive fruits, fruit particles or residues	Phenolic compounds	Mixture of oils with olive fruit materials and acids. Preferable conditions are 10:1 ^a^~10:3 ^a^, 0.5~5 wt % acid addition in oil-olive fruits mixtures, 90~100 °C, 90 min.	[42]
Olive oils	Olives	Flavouring agents	A crushing and malaxation process at 10~50 °C yielded flavoured oils with better antioxidant activity and high flavour stability.	[43]
Extra virgin olive oil	*Mentha piperita* L. and *Thymus mastichina* L.	Essential oils	Enriched with steam-distilled essential oils. A high level (0.008 mg/kg) of *Thymus* essential oil performed best in sensory test but *Mentha* essential oil must be kept at a low level (0.002 mg/kg).	[44]
Vegetable oils	Shrimp waste (*Panaeus indicus*)	Carotenoids	The carotenoid extraction using refined sunflower oil yielded higher than other oils under optimized conditions (2:1 ^a^, 150 min, 70 °C).	[45]
Virgin olive oil	Dried hot pepper, garlic, oregano and rosemary	Aromatic compounds	Flavoured oils with spice oily extracts (5:1 ^a^, 25 °C, dark, daily shaking) improved the oil stability. Tasters were able to distinguish among addition levels, and oils flavoured with 20 g/L of rosemary, 40 g/L of hot pepper, 40 g/L of oregano and 30 g/L of garlic were preferred at the end of the storage.	[46]
Palm, olive & sunflower oils	Olive leaf (*Olea europaea*)	Phenolic compounds	Enriched with methanol extract (500:1 ^b^ or 250:1 ^b^, 20 min shaking, 15 min sonication). Polyphenol intake by consuming French fries pan-fried in the enriched oils was 6~31 times higher than French fries fried in commercial oils, which is dependent on the frying oil type.	[47]
Refined edible oils	Microalgae (*Haematococcus pluvialis*)	Carotenoids	Carotenoid stock solutions (5 wt %) were prepared using acetone extract for oil enrichment. Palm oil was effective in retaining 90% of astaxanthin at 90 °C for 8 h without any changes in its ester form. Carotenoid loss was significant (60~90%) without changes in the fatty acid profile of the edible oils at 120 and 150 °C.	[48]
Palm oil	Giant tiger shrimp (*Panaeus mondon*)	Astaxanthin	Palm oil was used as solvents to extract carotenoids from shrimp waste (6:1 ^a^) at various particle sizes and temperatures.	[49]
Soybean, corn, grapeseed and olive oils	Microalgae (*Haematococcus pluvialis*)	Astaxanthin	Vegetable oils enriched directly with microalgae culture (1:1 ^b^, 25 °C, 48 h, stirring), the mean recovery yield was over 87.5%.	[50]
Extra virgin olive oil	Tunisian aromatic plants (thyme, rosemary, lavender, basil, lemon zests, white sage, garlic, menthe and geranium)	Aromatic compounds	The incorporation of some Mediterranean aromatic plants into olive oil relatively helped to improve their thermal resistance and stability. This may be due to the abundance of natural antioxidants, which were transferred into olive oils during the maceration process.	[51]
Refined corn oil	*Citrus aurantium* peel	Essential oil	Flavoured oil (8:3 ^a^, 1 h, 20 °C, 100 rpm) showed the highest total volatiles with unchanged fatty acid composition.	[52]
Flaxseed oil	Shrimp by-product (*Litopenaeus setiferus*)	Astaxanthin	Stirring solid-liquid enrichment (1:1 *w*/*w*, 60 min, 60 °C) yielded oils with better resistance to oxidation and temperature. By-products generated from shrimp peeling operations are a good source of high quality astaxanthin, which can be used as natural colorants and antioxidant ingredients in human food and other industrial applications.	[53]
Virgin olive oil	Olive cakes	Phenolic compounds	Enriched oils with extracts using different methods, in which oils with extracts from vegetative water and solid residue showed better quality than that with extracts from freeze-dried olive cakes.	[54]
Virgin olive oil	Freeze-dried olive cakes	Phenolic compounds	The enriched oil with extracts (7 mg/mL oil) of accelerated solvent extraction showed better oxidative stability, longer shelf life and less peroxides.	[55]
Refined sunflower oil	Olive pomace	Phenolic compounds	Such enriched oil (1:1 ^b^, 30 min) could decrease the degradation of lipidic components of the unsaponifiable fraction so as to improve stability.	[56]
Corn oil	Thyme flowers (*Thymus capitatus*)	Pigments, antioxidants	Flavoured oils (8:1 ^a^, 25 min agitation) showed improved thermal stability after heating than refined corn oil.	[57]
High oleic sunflower oil	Olive pomace	Phenolic compounds	The enriched oil with ethanolic extracts up to 400 µg/mL performed the best oxidation resistance during the frying process.	[58]
Refined edible oils	Olive pomace and leaves	Phenolic compounds	Oils mixing with ethanolic extracts up to favoured concentration of 200 or 400 μg/mL had similar profile to extra virgin olive oils.	[59]
Virgin olive oil	Rosemary, thyme & oregano	Antioxidants	Enrichment under stirring (20:1 ^a^, 25 °C or 35~40 °C) led to more efficient mass transfer than conventional maceration. A greatest enrichment of rosmarinic acids in oils was found for oregano.	[60]
Canola frying oil	Olive and hazelnut leaf, hazelnut green leaf cover	Phenolic compounds	Enriched with aqueous ethanolic extracts at 200 ppm phenolic equivalence level (100 °C, 8000 rpm, 7 min) to enhance thermo-oxidative stability without sensory quality deterioration.	[61]
Sicilian virgin olive oil	Sicilian olive samples from eight different cultivars	Phenolic compounds	Influence of olive variety and elevation of orchards on the phenolic compound content of Sicilian virgin olive oils (VOOs) was investigated, as well as the effect of VOO phenolic extracts on osteoblast cell growth using the human MG-63 osteosarcoma cell line. Olive oil phenolic content and its effect on human osteosarcoma cell proliferation varied according to the type of cultivar and grove altitude.	[62]
Olive oil	*Rubus* species (*Rubus ulmifolius*, *Rubus idaeus, Rubus caesius, Rubus saxatilis*, from the group *Rubus ulmifolius* preferably *Rubus ulmifolius frutticosus* Schott.)	Phenolic compounds	The invention relates to the use of an oily extract of plants of *Rubus* species and the topical treatment of cutaneous and mucosal pathologies and skin lesions. The invention further concerns a method for obtaining such extract through extraction in oils at room temperature.	[63]
Olive oil	Polyphenol compounds extracted from the olive cake	Phenolic-rich extract (oleuropein complex or secoiridoids: 89.4%; hydroxytyrosol, tyrosol and phenyl alcohols (vanillic acid, *p*-coumaric acid and vanillin) 3.5 %; and flavonoids, 6.0 %), obtained from the olive cake	High-polyphenol content functional virgin olive oil (FVOO) enriched with its own polyphenols, improved endothelial function in pre- and hypertensive subjects beyond the effects observed after the intake of a standard virgin olive oil (VOO) with moderate polyphenol content, in a postprandial randomised, cross-over, controlled trial.	[64]
Corn oil	Thyme dried flowers (*Thymus capitatus*)	Phenolic compounds, antioxidants	Antioxidant activities of the thyme-enriched oil were mainly due to the presence of phenolic compounds such as thymol and hydrocarbons such as γ-terpinene and *p*-cymene. The thyme-enriched oil could be considered as a new and natural source of antioxidant.	[65]
Virgin olive oil	Olive and thyme polyphenols	Phenolic compounds	The effects of virgin olive oil (VOO) enriched with its own phenolic compounds (PC) and/or thyme PC on the protection against oxidative DNA damage and antioxidant endogenous enzymatic system (AEES) were estimated in 33 hyperlipidemic subjects after the consumption of VOO, VOO enriched with its own PC (FVOO), or VOO complemented with thyme PC (FVOOT). The sustained intake of a FVOOT improves DNA protection against oxidation and AEES probably due to a greater bioavailability of thyme PC in hyperlipidemic subjects.	[66]

^a^ Oil to solid material ratio (mL/g); ^b^ Oil to liquid extracts ratio (mL/mL).

**Table 2:** Innovative techniques applied in the extraction of bio-active compounds using vegetable oils as solvents.

Technique	Matrix	Experimental Remarks	Reference
**Ultrasound**	Olive leaves (*Olea europaea* L.)	Solid-liquid oil enrichment (10:1 ^a^, 20 min, 25 °C) assisted by ultrasound (225 W, 50% amplitude, duty cycle 0.5 s) produced edible oils with better quality than non-ultrasonicated oils.	[113]
	Basil leaves (*Ocimum basilicum* L.)	Ultrasound-assisted aromatisation of 1L of olive oil with fresh basil leaves of different amounts. The essential oil contained in the basil leaves was directly extracted into the olive oil without any intermediate stage, which led to an aromatised olive oil in few minutes compared to several hours required in the conventional maceration.	[114]
	Olive leaves (*Olea europaea* L.)	Olive oil enrichment with phenolic compounds (e.g., oleuropein) from olive leaves by ultrasonic maceration (60 W, 16 °C and 45 min). The highest total phenolic content (414.3 ± 3.2 mg of oleuropein equivalent/kg of oil), oleuropein (111.0 ± 2.2 mg/kg of oil) and α-tocopherol (55.0 ± 2.1 g/kg of oil) concentrations obtained by optimized ultrasound-assisted extraction proved its efficiency compared to the conventional solid-liquid extraction.	[115]
	Sea buckthorn pomace (*Hippophae rhamnoides*)	Ultrasound-assisted extraction (power 0.67 W/g oil and 35 °C) has been used to greatly improve the direct enrichment of edible oils (sunflower, rapeseed, olive, and soya) with carotenoids from sea buckthorn pomaces in terms of quantity and process time (from 33.83 mg/L extract in 90 min obtained by conventional extraction to 51.64 mg/L extract in only 20 min by ultrasound).	[116]
	Carrot (*Daucus carota* L.)	Ultrasound-assisted extraction (carrot/oil ratio 2:10, 22.5 W, 40 °C and 20 min) using sunflower as alternative solvent to hexane obtained highest β-carotene yield (334.75 mg/L) in 20 min, while conventional solvent extraction obtained a similar yield (321.35 mg/L) in 60 min.	[82]
	Carrot residue (obtained after juice extraction) (*Daucus carota* L.)	Extraction using ultrasonic horn (20:0.3 ^a^, 100 W, 50 min, 50 °C), the maximum extraction yield of β-carotene was 83.32% while that was 64.66% when using ultrasonic bath.	[117]
	Pomegranate peels (*Punica granatum* L.)	Sunflower and soy oil were used as alternative solvents to study the effect of various parameters on the yield between ultrasound and conventional extraction, in which the optimal conditions for achieving maximum yield of carotenoids from pomegranate peels were 10:1 ^a^, 30 min, 51.5 °C, 58.8% of amplitude level and sunflower oil solvent.	[118]
**Microwave coupling ultrasound**	Vegetables, herbs, spices or fruits	Time-saving aromatizations of olive oil with different compounds from various plants were improved by ultrasound and microwave. The resulting flavoured oils are increasingly appreciated by European consumers.	[34]
	Sweet Pepper (*Capsicum annuum*)	Compared to traditional infusion or maceration (10:1 ^a^, 7 days), For the ultrasonic treatment, samples of olive oil were prepared by adding 10% and 20% dried chili pepper and subjected to ultrasound-extraction for 10 or 20 min. For microwave extraction, samples were added with 20% chili powder and treated for 10, 30 or 60 s. The production of flavored olive oils by using technologies such as microwave and ultrasound-extraction could allow the production of high quality oils, with fast and cost-effectively methods.	[119]
**Microwaves**	Aromatic plants	A patented method for the extraction of aromas from aromatic plants using microwave is disclosed.	[83]
	Olive leaf	Liquid-liquid enrichment with microwave phenolic extract (1:1 ^a^, 15 min, 600 units/min). Olive oil was the most enriched. Enrich oils obtained a better taste quality.	[120]
	Olive waste (orujo)	Solid-liquid and liquid-liquid oil enrichments (1:1 ^a^, 30 min) with dilutions of microwave phenolic extracts. The phenol distribution factor increases with high level of unsaturated fatty acids whereas high-saturated fatty acid content decreases this factor.	[121]
	Fresh vegetable materials	The present invention relates to a method for obtaining an oily extract of plants from plant material comprising the steps of mixing the plant material with a fat, heating (microwave at 0.1 to 5 W/g of oil and plant material mixture) said mixture and recovery of the oily extract. The present invention finds particular application in the field of the production of special extracts, scent extracts, extracts perfuming, supply of raw materials, olfactory raw materials, active ingredients, for example in cosmetics and/or dermatology.	[122]
	Daylily (*Hemerocallis fulva*)	The invention relates to the use of an oily composition comprising a lipophilic extract of daylily as an active ingredient for the preparation of a topical cosmetic composition intended to improve complexion radiance and/or to even skin tone, to a non-therapeutic cosmetic skin treatment method using such a cosmetic composition. The extract is obtained by the technique of heating by microwaves using avocado oil and rose hip oil.	[123]
**Supercritical fluids**	Tomato (*Solanum lycopersicum* L.)	Extraction of lycopene from tomato using SC-CO_2_ extraction in the presence of vegetable oil as co-solvent. The pre-treatment of raw material (drying, grinding and screening) is necessary in order to obtain significative yields of the extractable lycopene. The operative parameters (flow, time, pressure, etc.) are also crucial for better yields and the best operative conditions found are the following: pressure 450 bar, temperature 65–70 °C, CO_2_ flow rate 18–20 kg CO_2_/h, average particle sizes of the material of about 1 mm, presence of a vegetable oil as co-solvent (about 10%).	[97]
	Red pepper (*Capsicum frutescens* L.)	Enriched oils with supercritical CO_2_ extracts (0.5 wt %) at low pressure and velocity (40 °C, 10 min) performed stable.	[124]
	Carrot (*Daucus carota* L.)	Employing canola oil as a continuous co-solvent in SC-CO_2_ extraction is a novel and efficient technique for the recovery of carotenoids from natural materials.	[95]
	Microalgae (*Haematococcus pluvialis*)	Soybean oil and olive oil used as co-solvents were investigated for SC-CO_2_ extraction of astaxanthin from *H. pluvialis*. The result of the SC-CO_2_ extraction of astaxanthin with the presence of 10% by volume olive oil showed the higher increase in the amount of astaxanthin extracted.	[112]
	Marigold (*Tagetes erecta* L.)	SC-CO_2_ extraction of lutein esters from marigold with soybean oil as a co-solvent was performed. Results showed that the data could be well fitted to a second-order polynomial model with a R^2^-value of 0.9398. The model predicted that the optimal conditions were 35.5 MPa, 58.7 °C, CO_2_ flow rate of 19.9 L/h with 6.9% of soybean oil as a co-solvent, and under such conditions, the maximum yield of 1.04 g lutein/100 g marigold could be achieved.	[100]
	Marigold (*Tagetes erecta* L.)	Medium-chain triglycerides (MCTs), sunflower seed oil, soybean oil, rapeseed oil and n-hexane were used as co-solvents to promote supercritical carbon dioxide (SC-CO_2_) extraction of lutein esters from marigold (*Tagetes erecta* L.). The optimum extraction conditions within the experimental range were predicted to be: extraction pressure of 46.8 MPa, temperature of 65.9 °C and MCT concentration of 1.5% (*w*/*w* of CO_2_), with a CO_2_ flow rate of 10 kg/h and extraction time of 3 h. The maximum yield of lutein esters under these conditions was predicted to be 1.3 g/100 g marigold.	[101]

^a^ Oil to solid material ratio (mL/g); ^b^ Oil to liquid extracts ratio (mL/mL).
